# Molecular mechanisms of cisplatin resistance in ovarian cancer

**DOI:** 10.1016/j.gendis.2023.06.032

**Published:** 2023-08-02

**Authors:** Chenying Jiang, Chenjun Shen, Maowei Ni, Lili Huang, Hongtao Hu, Qinhui Dai, Huajun Zhao, Zhihui Zhu

**Affiliations:** a School of Pharmaceutical Sciences, Zhejiang Chinese Medical University, Hangzhou, Zhejiang 311402, China; b The Cancer Hospital of the University of Chinese Academy of Sciences (Zhejiang Cancer Hospital), Institute of Basic Medicine and Cancer (IBMC), Chinese Academy of Sciences, Hangzhou, Zhejiang 310005, China

**Keywords:** Cisplatin, Drug resistance, Molecular mechanisms, Ovarian cancer, Therapeutic strategies

## Abstract

Ovarian cancer is one of the most common malignant tumors of the female reproductive system. The majority of patients with advanced ovarian cancer are mainly treated with cisplatin-based chemotherapy. As the most widely used first-line anti-neoplastic drug, cisplatin produces therapeutic effects through multiple mechanisms. However, during clinical treatment, cisplatin resistance has gradually emerged, representing a challenge for patient outcome improvement. The mechanism of cisplatin resistance, while known to be complex and involve many processes, remains unclear. We hope to provide a new direction for pre-clinical and clinical studies through this review on the mechanism of ovarian cancer cisplatin resistance and methods to overcome drug resistance.

## Introduction

Ovarian cancer is the seventh most commonly diagnosed cancer among women worldwide.^1^ According to the international unified classification established by the World Health Organization, ovarian cancer is divided into many categories from different histological sources, including epithelial tumors, germ cell tumors, sex cord interstitial tumors, and metastatic tumors.^2^ Epithelial ovarian cancer is the most common type, representing about 70% of ovarian malignant tumors, and is more common in middle-aged and old women and less common in pre-adolescent women.^3^ There are many pathological types of epithelial ovarian cancer, among which serous adenocarcinoma accounts for 70%; other types include endometrioid adenocarcinoma, clear-cell carcinoma, and mucinous adenocarcinoma.^3^ The incidence of ovarian cancer is lower than that of cervical cancer and endometrial cancer, which ranks third among female reproductive system malignancies, but its mortality ranks first.^4^ According to the American Cancer Society, it is estimated that 13,270 deaths were a result of ovarian cancer in the United States in 2023.^4^ Since the mid-1970s, the mortality of ovarian cancer has decreased by more than 30% due to diminutions in incidence and treatment improvements.^5^ However, early-stage ovarian cancer is not usually diagnosed because of a lack of specific early symptoms and effective screening strategies. About 70% of patients diagnosed with International Federation of Gynecology and Obstetrics stage III or IV ovarian cancer will experience a recurrence after treatment.^6^ It is the biggest barrier, with a 49.7% 5-year survival rate after diagnosis.^7^

As the most successful metallic anti-cancer drug, platinum shows efficient and broad-spectrum anti-tumor activities that play an important role in clinical cancer treatment.^8^ Cisplatin was the first and the most effective platinum-based anti-neoplastic drug.^9^ It is used for various solid cancers, including ovarian, cervical, bladder, and lung cancer.^10^ For many years, tumor cytoreductive surgery and combination chemotherapy based on cisplatin and paclitaxel have been the main treatment options for ovarian cancer.^11^ However, in the course of clinical treatment, cisplatin toxicity and resistance have gradually emerged as a challenge to improving the prognosis of patients.

Tumor-free interval is an important indicator of the sensitivity of chemotherapy. Patients are traditionally labeled as either platinum-sensitive (relapse >6 months since last platinum therapy) or platinum-resistant (relapse <6 months since last platinum therapy).^12^ Cisplatin resistance not only leads to decreased sensitivity of chemotherapeutic drugs to ovarian cancer but results in a detrimental effect on treatments. Studies have found that cisplatin resistance in ovarian cancer patients may be associated with multiple factors, including intracellular drug accumulation, cell metabolism, and DNA damage repair. In recent years, efforts have been made to develop various drugs and use new technologies to reverse cisplatin resistance. In this review, we briefly summarize the mechanism of cisplatin resistance in ovarian cancer and some strategies to overcome this resistance or restore its sensitivity, which we hope will be helpful in the treatment of ovarian cancer in the future.

## Mechanisms of cisplatin-based chemotherapy resistance in ovarian cancer

The biggest drawback of cisplatin-based chemotherapy is ovarian cancer cell resistance. Many patients with ovarian cancer relapse due to cisplatin resistance. Cisplatin resistance may be caused by lowering intracellular drug accu-mulation, detoxification of glutathione (GSH), and repairing DNA.^13^, ^14^, ^15^ In this review, cisplatin resistance occurs at the following five stages: before cisplatin's entry into tumor cells, during the influx and efflux of cisplatin through the cell membrane, in the presence of cisplatin in the cytoplasm, during the interaction of cisplatin with nuclear DNA, and after post-targeting.

## Before cisplatin's entry into tumor cells

Blood circulation is a major obstacle prior to cisplatin's entry into cells. A large amount of cisplatin administered intravenously to patients is rapidly bound by sulfhydryl-containing proteins such as blood plasma proteins, transporters, and cysteine, particularly human serum albumin.^16^ The binding of cisplatin to human serum albumin reduces the anti-tumor activity of cisplatin.^17^

As a critical cell-cell communication medium, extracellular vesicles help regulate tumor metastasis and proliferation, including apoptotic bodies, microvesicles, and exosomes.^18^ It has been reported that human mesenchymal stem cell derived-extracellular vesicles regulate cisplatin resistance in ovarian cancer cells by releasing miR-18a-5p.^19^ In addition, a study focused on the expression of circFoxp1 in the serum of ovarian cancer found that the expression of exosomal circFoxp1 in cisplatin-resistant patients was significantly higher than that in cisplatin-sensitive patients,^20^ suggesting the possibility of using other circulating exosomes as biomarkers and potential therapeutic targets for ovarian cancer.

The extracellular matrix is an important part of the milieu surrounding cells. Matrix metalloproteinases (MMPs) can degrade various proteins in the extracellular matrix and are currently being studied as potential therapeutic targets in cancer cells. The relationship between progression, invasiveness, and metastatic capacity of ovarian cancer and the activity of MMPs has been described.^21^ Wang et al^22^ found increased MMP-19 and MMP-20 expression were correlated with platinum resistance. MMP-2 and MMP-9 are frequently identified as potential biomarkers of chemoresistance in ovarian cancer.^23^ Matrix adhesion is also implicated in resistance mechanisms. One study has shown that cisplatin resistance can be enhanced by the specific stiffness of extracellular matrix substrates through focal adhesion kinase and β1 integrin-phosphorylated myosin light chain-yes-associated protein signaling, while inhibiting apoptosis.^24^ Moreover, cisplatin resistance of ovarian cancer cells can be significantly increased due to overexpression of collagen VI, an extracellular matrix protein with growth factor-like characteristics.^25^

## During the influx and efflux of cisplatin through the cell membrane

**Figure 1:**
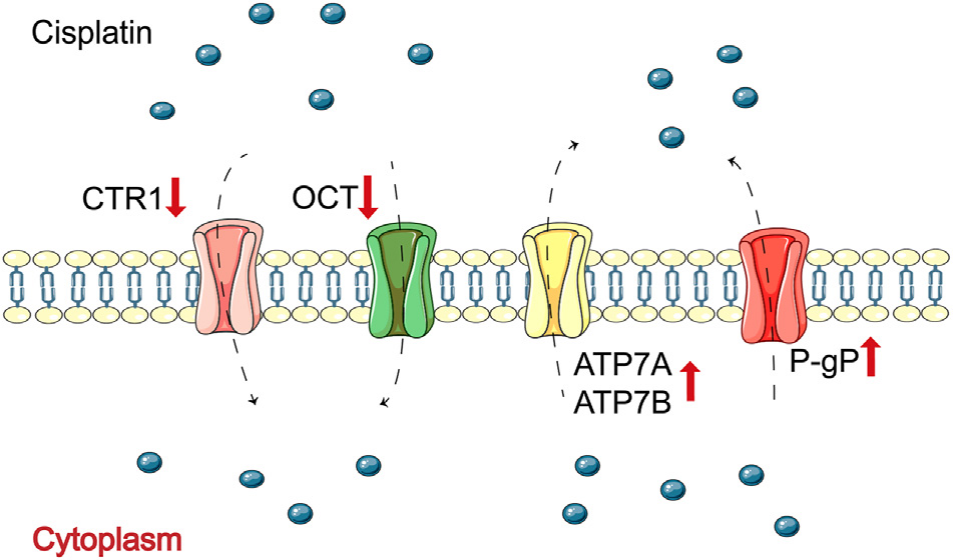
Influx and efflux of cisplatin through the cell membrane. The interaction of cisplatin with membrane proteins has been shown to affect the influx and efflux of cisplatin.

Before entering the cell, interactions between cisplatin and membrane proteins, lipids, and other biomolecules affect the influx and efflux of cisplatin, resulting in changes in intracellular cisplatin accumulation, which can result in poor therapeutic effects and cause cisplatin resistance (Fig. 1).

First, the lower accumulation of intracellular cisplatin may be related to reduced drug uptake. Passive diffusion and transport proteins are the primary actors of cisplatin influx. As permeable enzymes located on the cell membrane, copper transporters regulate cellular copper homeostasis.^26^ High copper transporter 1 expression has been reported in advanced ovarian cancer with elevated survival of patients and improved cisplatin sensitivity.^27^ Solute carriers, as membrane transporters, play a role in cisplatin resistance to ovarian cancer.^28^ Nearly 30 kinds of solute carriers are involved in chemotherapy resistance; some are down-regulated in cisplatin-resistant ovarian cancer cells while others are not.^29^ In addition, organic cation transporters facilitate cisplatin's entry into cells; their down-regulation reduces cisplatin nephrotoxicity^30^ and delays cisplatin incorporation.^31^

The drug efflux system is also an important reason for decreased effective cisplatin accumulation in tumor cells, meaning that cisplatin is partly pumped out before reaching its intracellular target. Copper-transporting adenosine triphosphatases (ATPases), especially ATP7A and ATP7B, mediate the excretion of cisplatin.^32^ High expression of ATP7A and ATP7B are correlated with an inferior response to cisplatin, thus inducing the development of cisplatin resistance in ovarian cancer.^33^ Multidrug resistance protein 1, also known as P-glycoprotein, acts as an efflux pump, which outputs substrates to cells using energy from ATP hydrolysis.^34^ Overexpression of multidrug resistance protein 1 is also one of the main factors of cisplatin resistance in ovarian cancer.^35^ At present, it is necessary to develop appropriate efflux pump inhibitors to weaken drug efflux, to reverse cisplatin resistance.

## In the presence of cisplatin in the cytoplasm

**Figure 2:**
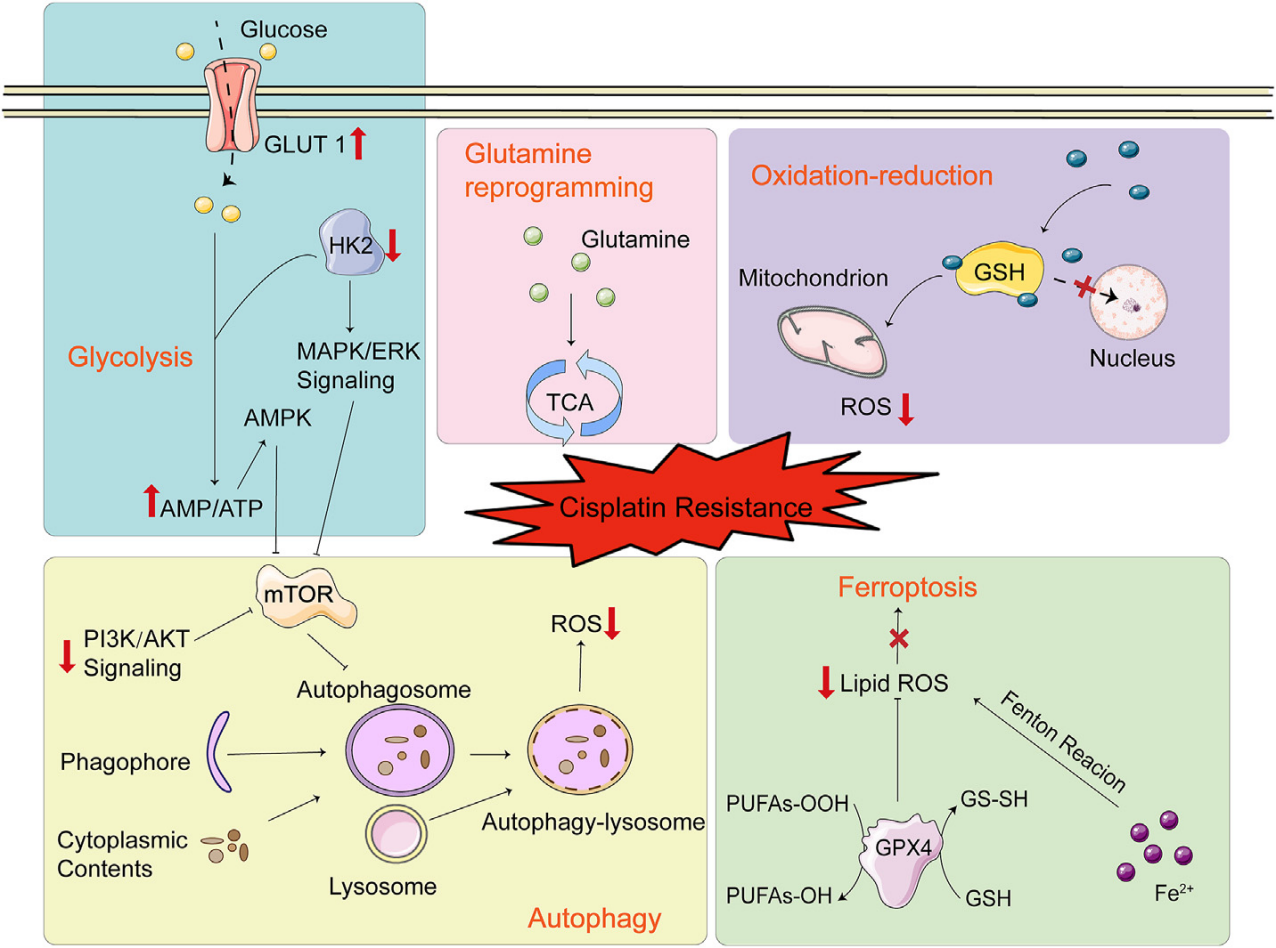
Presence of cisplatin in the cytoplasm. Oxidation-reduction, autophagy, ferroptosis-inhibition, and metabolic reprogramming impact the development of resistance in the presence of cisplatin in the cytoplasm.

Resistance of cisplatin in the cytoplasm is mainly related to GSH (Fig. 2). Due to its high affinity for cisplatin, GSH competitively inhibits the binding of cisplatin to DNA, leading to cisplatin resistance.^36^ A recent study showed that the level of GSH in cisplatin-resistant cell line A2780 (A2780CIS) is much higher than that in A2780 cells.^37^

Increased reactive oxygen species (ROS) generation leads to oxidative damage of biological molecules including DNA, proteins, and lipids, likely resulting in apoptosis.^38^ Mitochondria are important for cisplatin sensitivity. Cisplatin-resistant ovarian cancer cells contain lower levels of mitochondrial ROS (mtROS) compared with cisplatin-sensitive cells.^39^ Cisplatin-induced cytotoxicity is closely related to mtROS production. In addition, the existence of antioxidants such as GSH and GSH peroxidase in mitochondria may also be important in cisplatin resistance.^40^ Overexpression of GSH not only inactivates cisplatin but also reduces sensitivity to cisplatin by reducing mtROS.^41^ Han et al^42^ found that mitochondrial fission and cisplatin resistance were promoted in ovarian cancer cells under hypoxic conditions. However, the level of endogenous ROS in cisplatin-resistant ovarian cancer cells is higher than that in cisplatin-sensitive cells.^43^ The complicated mechanisms of ROS regulation affecting cisplatin resistance are still controversial and will require further exploration.

Autophagy is a complicated metabolic pathway to achieve the renewal of organelles and the stability of the intracellular environment.^44^ Autophagy is the core of the cellular stress response.^45^ For tumor cells, autophagy can provide energy for the body, thus protecting chemotherapy-resistant cancer cells. The phosphatidylinositol-3-kinase, protein kinase B, and mammalian target of rapamycin signaling pathway is a major regulator of autophagy and associated with chemoresistance in cancer.^46^ After phosphatidylinositol-3-kinase activation, it stimulates its downstream target protein kinase B to activate mammalian target of rapamycin, finally inhibiting autophagy and improving the sensitivity of the human multidrug-resistant phenotype ovarian cancer cell line SKVCR.^47^

Ferroptosis is an iron-dependent new programmed necrosis, which differs from apoptosis, necrosis, and autophagy. The main mechanism of ferroptosis is that under the action of excessive iron or ester oxygenase and oxidative stress, unsaturated fatty acids on cell membrane are catalyzed to induce lipid peroxidation; in addition, the expression of the antioxidant system, GSH and glutathione peroxidase 4, is decreased.^48^ This mechanism has been reported to reduce cisplatin resistance in ovarian cancer by increasing lipid metabolism activity and driving redox-induced ferroptosis.^49^^,^^50^ Combination therapy based on cisplatin and the ferroptosis inducer erastin appears to minimize ovarian cancer cell growth and overcome resistance.^51^

Metabolic reprogramming refers to the metabolic changes made by cells in response to a variety of pressures. Metabolic reprogramming is prevalent in many diseases, such as glucose metabolism, amino acid metabolism, and other metabolic pathways. The metabolism of cancer cells is quite different from that of normal and healthy cells. Cancer cells are more metabolically active and have a higher reproduction rate.^52^ The Warburg effect proposes that in addition to normal mitochondrial respiration, glycolysis is crucial in cancer cells for much more energy production.^53^ Existing evidence suggests enhanced glycolysis can induce either intrinsic or acquired resistance to cisplatin in several cancer cell types.^54^^,^^55^ As a major transmembrane transporter for maintaining efficient glucose transport, glucose transporter 1 is widely expressed in human tissues and organs. Inhibitors of glucose transporter 1 (STF 31, IOM-1190) attenuated the proliferation of cisplatin-resistant high-grade serous ovarian cancer cells.^56^ Hexokinase 2 catalyzes glucose phosphorylation in glucose-6-phosphate, the rate-limiting step in glycolysis. Hexokinase 2 can promote cisplatin resistance in ovarian cancer by enhancing extracellular regulated protein kinase-mediated autophagy.^57^

As a classical non-essential amino acid, glutamine plays a crucial role in tumor cell growth and proliferation.^58^ Through the tricarboxylic acid cycle, reprogramming glutamine plays a nutritional role in survival and contributes to the development of chemotherapy resistance in tumor cells.^59^ Cisplatin-resistant ovarian cells were more sensitive to killing due to glutamine deprivation.^60^ Therefore, inhibition of the glutamine metabolism pathway, which is associated with a poor prognosis of ovarian cancer, may become a new strategy for treating drug-resistant ovarian cancer.^61^

## Interaction of cisplatin with nuclear DNA

### Metallothionein

DNA is the primary binding target of cisplatin therapy. Restricting DNA binding to cisplatin can lead to drug resistance. After cisplatin enters the nucleus, it binds to cysteine thiols of metallothionein due to its transition metal core, resulting in a loss of activity. As a low molecular weight protein, metallothionein plays an important role in the homeostasis and detoxification of metal ions.^62^ It has been reported to be one of the causes of tumor cell resistance to cisplatin, which is unfavorable for patients' treatment.^63^ Besides, higher nuclear metallothionein expression has been observed in A2780CIS cells compared with cisplatin-sensitive cells.^64^

### DNA repair

**Figure 3:**
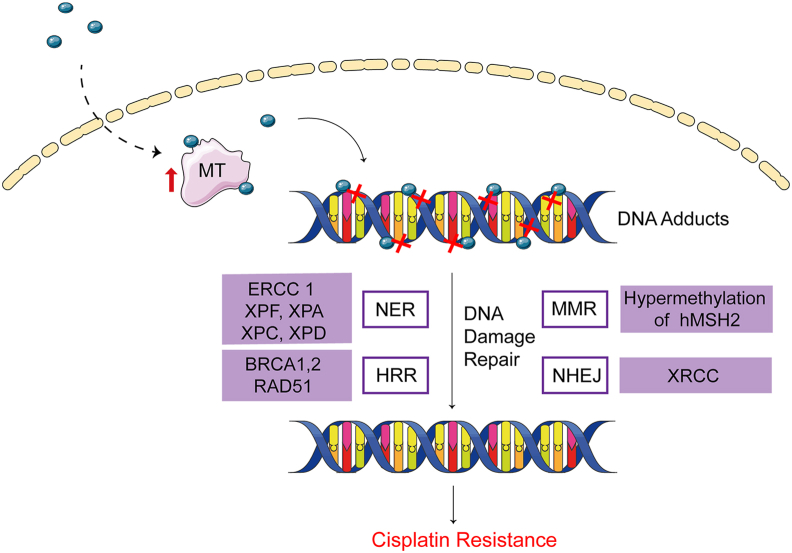
Interaction of cisplatin with nuclear DNA. MT competitively binds to cisplatin after cisplatin enters the nucleus, resulting in reduced cisplatin-DNA interaction. DNA adducts restore the normal structure and confer resistance under the DNA damage repair systems, including NER, MMR, HRR, and NHEJ.

DNA repair is the most important cause of cisplatin resistance, mainly including nucleotide excision repair (NER), homologous recombination repair (HRR), mismatch repair (MMR), and nonhomologous end joining (NHEJ) (Fig. 3).^65^

NER aims to eliminate the damage of helical twisted DNA. NER can avoid DNA damage caused by ultraviolet radiation and electrophilic compounds, such as environmental carcinogens and cancer chemotherapy drugs.^66^ The changes in NER-associated genes in ovarian cancer can improve the overall survival and progression-free survival of cisplatin-resistant patients.^67^ Therefore, the mRNA and protein levels of NER-related components, such as excision repair cross-complementing group genes and xeroderma pigmentosum (XP) group genes, have been quantified to investigate the relationship between NER and cisplatin resistance mechanisms in ovarian cancer and evaluate their prognostic value for patients.^68^ Du et al^69^ found that the cisplatin sensitivity of cisplatin-resistant cell line SKOV-3 (SKOV-3CIS) could be remarkably increased by excision repair cross-complementing group 1 gene-specific silencing. XPF, in combination with excision repair cross-complementing group 1, can cleave bulky DNA lesions and improve cisplatin resistance in A2780CIS cells.^70^ XPA is an important scaffold for NER, which has a high affinity with damaged DNA. XPD is a key member of the human general transcription factor IIH complex. Higher expression of XPA and lower expression of XPD protein levels have been observed in cisplatin-sensitive metastatic ovarian cancer cells.^71^^,^^72^ XPC is an essential DNA damage recognition protein.^73^ In the XPC gene, three single nucleotide polymorphisms were correlated with prolonged progression-free survival in advanced cisplatin-resistant ovarian cancer patients.^74^ These studies underscored the importance of these genes or proteins that may become reliable indicators of cisplatin resistance.

HRR is critical for the repair of double-strand breaks in the S and G2 phases of the cell cycle and includes some vital proteins and genes such as RAD51 recombinase and breast cancer early onset gene 1/2.^75^^,^^76^ In HRR, 3′ single-stranded DNA tails are formed by nucleolytic resection of DNA ends, which are used for strand invasion. Then, the double-stranded damaged segment is paired with a homologous DNA template to restore the chromosome integrity.^77^ Germline mutations of breast cancer early onset genes have a profound impact on the efficacy of cisplatin and survival in ovarian cancer patients. Patients carrying mutations may be more likely to respond to cyclical cisplatin-based treatment.^78^ The cytotoxicity of cisplatin can be enhanced and the sensitivity of cisplatin-resistant cells can be restored by silencing breast cancer early onset gene 1/2 pathway genes.^79^ As a recombinase, RAD51 participates in the double-strand repair of HRR, which can be a viable marker to predict the response to cisplatin-based chemotherapy due to its overexpression in cisplatin-resistant ovarian cancer cells.^80^

MMR can correct base mismatches during DNA replication. MMR deficiency affects cisplatin sensitivity. An early study showed that MMR depended on epigenetic modifications.^65^ Hypermethylation of MMR gene *hMSH2* enhanced the sensitivity of ovarian cancer cells to cisplatin.^81^

Differing from HRR, the speed of repair is faster, but some sequences may be deleted during NHEJ.^82^ X-ray repair cross-complementing genes in the NHEJ signaling have an effect on double-strand breaks caused by ionizing radiation.^83^ However, current studies have been limited to the relationship between X-ray repair cross-complementing gene expression and the prognosis of cisplatin chemotherapy in ovarian cancer. No specific mechanism of X-ray repair cross-complementing genes for cisplatin resistance has been reported.^84^

## Resistance after post-targeting

### Non-coding RNAs (ncRNAs)

NcRNAs refer to RNAs without coding potential. MicroRNAs (miRNAs), long non-coding RNAs (lncRNAs), and circular RNAs (circRNAs) have been proposed as novel research targets in addressing ovarian cancer resistance^85^^,^^86^ (Table 1).

**Table 1:** Non-coding RNAs regulate cisplatin resistance in ovarian cancer.

Type	Gene name	Main target gene	Function	Reference
MiRNA	miR-29	COL1A1	Cisplatin sensitivity	88
	miR-216b	PARP1	Cisplatin sensitivity	89
	miR-378a-3p	MAPK1, GRB2	Cisplatin sensitivity	90
	miR-130a	XIAP	Cisplatin sensitivity	91
	miR-146b	FBXL10	Cisplatin sensitivity	92
	miR-133b, miR-186	MDR1, GST-π	Cisplatin sensitivity	93, 94
	miR-132	BMI-1	Cisplatin sensitivity	95
	miR-302	ATAD2	Cisplatin sensitivity	96
	miR-152, miR-185	DNMT1	Cisplatin sensitivity	97
	miR-149–3p	CDKN1A, TIMP2	Cisplatin resistance	98
LncRNA	MCF2L-AS1	IGF2BP1	Cisplatin resistance	100
	ACTA2-AS1	Wnt5a	Cisplatin resistance	101
	CASC10	NUP43	Cisplatin resistance	102
	HOXA11-AS	Beclin1, LC3-II/I	Cisplatin resistance	103
	WDFY3-AS2	SDC4	Cisplatin resistance	104
	CTSLP8	PKM2, c-Myc	Cisplatin resistance	105
	LINC01508	YAP	Cisplatin resistance	106
	PRKAR1B-AS2	PI3K, AKT, mTOR	Cisplatin resistance	107
	CERNA1	BCL2L10	Cisplatin sensitivity	108
	SLC25A21-AS1	KCNK4	Cisplatin sensitivity	109
CircRNA	circSnx12	SLC7A11	Cisplatin resistance	112
	circPBX3	IGF2BP2, ATP7A	Cisplatin resistance	113
	circTYMP1	TGF1B	Cisplatin resistance	114
	circCdr1as	SCAI	Cisplatin sensitivity	115
	circLPAR3	PDK1	Cisplatin resistance	116

MiRNAs mediate post-transcriptional gene silencing of target genes by targeting the 3′ untranslated region (3′ UTR) of mRNA.^87^ Down-regulation of miR-29, miR-216b, miR-378a-3p, and miR-130a conferred cisplatin resistance to ovarian cancer cells.^88^, ^89^, ^90^, ^91^, ^92^, ^93^, ^94^, ^95^, ^96^, ^97^ However, the down-regulation of miR-149–3p in ovarian cancer significantly inhibited cell migration and invasion but also improved the response to cisplatin.^98^ Confirming the miRNA targets that alter cisplatin sensitivity will be important in understanding the mechanisms of cisplatin resistance.

LncRNAs, non-coding RNAs with a length of more than 200 nucleotides, have been characterized as essential mediators of cell proliferation, invasion, apoptosis, metabolism, immune evasion, and chemoresistance in ovarian cancer cells.^99^ Several lncRNAs have been reported to be highly expressed in ovarian cancer and promote cisplatin resistance, such as MCF2L-AS1, ACTA2-AS1, and CASC10,^100^, ^101^, ^102^, ^103^, ^104^, ^105^, ^106^, ^107^ while other lncRNAs have been found to increase cisplatin sensitivity of ovarian cancer cells, including CERNA1 and SLC25A21-AS1.^108^^,^^109^

Different from traditional linear RNAs with 5′–3′ polar polyadenylation tails, circRNAs have a closed-loop structure, which is not affected by RNA nucleic acid exonucleases and thus exhibits more stable expression.^110^ CircRNAs can form a competing endogenous RNA (ceRNA) network with miRNAs, which regulates tumor proliferation, invasion, and chemosensitivity by directly or indirectly targeting genes.^111^ Compared with surrounding normal tissues, the expression of circRNAs in ovarian cancer cells may be up-regulated or down-regulated. Recent studies have gradually explored the functions of circRNAs in cisplatin resistance.^112^, ^113^, ^114^, ^115^, ^116^

A comprehensive evaluation of ncRNAs in cisplatin-resistant cells is necessary to determine the exact mechanism of cisplatin resistance in ovarian cancer. Current functional studies of ncRNAs have mainly focused on miRNAs and lncRNAs. However, little is known about the whole world of ncRNAs. Therefore, it will be important to further our understanding of the regulatory network of ncRNAs, that is, the interactions of all ncRNAs and their connections with DNA.

### Epithelial-mesenchymal transition

Epithelial-mesenchymal transition is a process in which cells gain invasive mesenchymal ability, which is closely related to cancer metastasis. Recent evidence suggests that epithelial-mesenchymal transition contributes to chemoresistance, and it is modulated by a number of transcription factors, such as SNAIL 1 and SNAIL 2.^117^ The primary function of SNAIL 1 and SNAIL 2 is to induce epithelial-mesenchymal transition by repressing E-cadherin transcription, although they can inhibit other epithelial markers (occluding) or tumor suppressors as well. Mesenchymal markers (e.g., vimentin), as well as the activity of metalloproteinases, are up-regulated simultaneously.^118^ When SNAIL 1 and SNAIL 2 are knocked down in A2780CIS cells, the epithelial-mesenchymal transition phenotype is largely reversed and cisplatin sensitivity is restored.^119^

### Tumor heterogeneity

Tumor heterogeneity is a characteristic of malignant tumors and is manifested by changes in molecular biology or genes, resulting in changes in proliferation, invasion, and drug sensitivity.^120^ Tumor heterogeneity may arise from genetic mutations or epigenetic or genetic modifications. DNA methylation, an important epigenetic modification in organisms, is catalyzed by DNA methyltransferases (DNMTs). As a member of the DNA methyltransferase family proteins, DNA methyltransferase 1 has methyltransferase activity.^121^ It is responsible for the accurate replication of DNA methylation. Xiang et al^97^ revealed that by directly targeting DNA methyltransferase 1, the sensitivity of cisplatin was changed both *in vitro* and *in vivo*.

Cancer stem cells are considered a determinant of intra-tumoral heterogeneity.^122^ As self-renewable pluripotent stem cells, ovarian cancer stem cells are more aggressive and induce disease recurrence, which has an essential role in cancer development, metastasis, and resistance to chemotherapy.^123^ The combination of clonal evolution and hierarchical ovarian cancer stem cell models is considered responsible for ovarian cancer development and recurrence. Identifying and functionally characterizing ovarian cancer stem cells will be essential for the development of effective therapies. Several established ovarian cancer stem cell markers, including CD44, CD117, CD133, CD24, and ALDH have been used to identify ovarian cancer stem cell populations.^124^ However, due to the lack of clinical samples from cisplatin-resistant ovarian cancer, it remains a limitation that most studies rely on tissues that did not receive chemotherapy.

### Cellular senescence

Cellular senescence is often a response to intrinsic or extrinsic stress, such as DNA damage in the human body. The presence of senescent cells prevents the tissue from continuously proliferating and undergoing self-repair. Previous studies have found that senescent cells can generate and release cytokines, which activate the remaining cancer cells to acquire drug resistance during chemotherapy.^125^

### Strategies to reverse cisplatin resistance in ovarian cancer

In recent years, various drugs have been developed according to the different mechanisms of cisplatin resistance. In addition to platinum drugs, cisplatin-resistant therapeutic drugs for ovarian cancer, which are widely applied in the clinic, mainly include anti-angiogenic agents, immunosuppressors, and poly (ADP-ribose) polymerase (PARP) inhibitors (Table 2). In addition, changing the dosage form and drug delivery system is also important in overcoming cisplatin resistance in ovarian cancer (Table 3).

**Table 2:** Clinical studies for the treatment of cisplatin resistance in ovarian cancer.

Type of regimen	Therapy	Research progress	Treatment-related indicators			Reference
			Progression-free survival (months)	Overall survival (months)	Objective response rate (%)	
Platinum alternative drugs	Carboplatin	In clinic	4.8			127
	Carboplatin, paclitaxel	In clinic	12.5	7.4		126
	Oxaliplatin	In clinic			23.0	129
	Paclitaxel	In clinic	36.5			132
	Paclitaxel, cisplatin	In clinic	30.0	58.5		132
	Paclitaxel, carboplatin	In clinic	25.0	55.0		132
	Topotecan	In clinic	4.8	15.8	10.0	136
	Pemetrexed	Phase II clinical trial	2.9	11.4	21	139
	Pemetrexed, carboplatin	Phase II clinical trial	9.4		32.8	140
	Gemcitabine	In clinic	3.0			141
	Topotecan, cisplatin	In clinic	2.5	19.3	22.6	142
	PLD (pegylated liposomal doxorubicin)	In clinic	3.7			143
	PLD, olaratumab	Phase II clinical trial	5.5			143
Anti-angiogenic drugs	Bevacizumab	In clinic	4.4			145
	Bevacizumab, gemcitabine	In clinic	4	15.3	25.0	148
	Ramucirumab	Phase II clinical trial	3.2	10.0		149
	Apatinib	Phase Ⅳ clinical trial	5.0	9.5		151
	Apatinib, etoposide	Phase II clinical trial	5.6	10.3	36.4	150
PARP inhibitors	Olaparib	In clinic	2.9		17.9	156
	Olaparib, PLD	Phase II clinical trial	5.8		29.0	155
	Niraparib	Phase Ⅲ clinical trial	21.9	20.1		157
	Rucaparib	Phase Ⅲ clinical trial	16.6			158
Cell-cycle kinase inhibitors	Ribociclib	Phase I clinical trial	11.4		79.3	163
	Abemaciclib	Phase Ⅲ clinical trial	5.3			165
	Abemaciclib, fulvestrant	Phase Ⅲ clinical trial	16.9	46.7		165
Immunotherapy	Nivolumab	Phase II clinical trial	3.5	20.0		172
	Nivolumab, ipilimumab	Phase II clinical trial	3.9	28.1	31.4	174
	Pembrolizumab	Phase II clinical trial	2.1		7.4	176
	Pembrolizumab, niraparib	Phase I/II clinical trial			18.0	177
	Pembrolizumab, PLD	Phase II clinical trial			19.0	180
	Pembrolizumab, bevacizumab, cyclophosphamide	Phase II clinical trial	10.0		47.5	181
	Pembrolizumab, cisplatin, gemcitabine	Phase II clinical trial	6.2	11.3	60.0	182
	Avelumab, PLD	Phase Ⅲ clinical trial	3.7	15.7		183
	Durvalumab, olaparib	Phase II clinical trial	5.36	15.5	42.9	184

**Table 3:** Role of drug delivery systems in reversing cisplatin resistance.

Type of regimen		Payload	Main effects	Reference
Nanoparticle delivery systems	Lipid-based delivery systems	Layer-by-layer polymeric liposomal nanoparticles	Facilitate the selective targeting of CD44 receptors to ovarian cancer	187
		Cross-linked multilamellar liposome vesicles	Reduce systemic toxicity; induce cell apoptosis	188
	Polymeric delivery systems	Polymer-polyamide-amine dendrimers	Enhance the nonspecific and targeted uptake of cisplatin in ovarian cancer cells.	189
		PLGA-PEG nanoparticles	Improve cisplatin uptake and stability; lower hepatotoxicity of wortmannin; block DNA repair and enhances the synergistic cytotoxicity of the drug combination in PROC	190
		MPEG-PLA nanoparticles	Reduce systemic toxicity *in vivo*; induce cell apoptosis	191
	Inorganic delivery systems	Magnetically Fe_3_O_4_ nanoparticles	Visualize the tumor site location; enhance cytotoxicity	193
		Hyaluronic acid-conjugated mesoporous silica (MSN-HAs) nanoparticles	Carry Si-TWIST into target cells and inhibit epithelial-mesenchymal transition by sustaining TWIST knockdown *in vitro*	194
	Virus-like nanoparticles	Tobacco mosaic virus	Superior cytotoxicity and double-strand breaks; biphasic release profiles	195
Exosomes		Umbilical cord blood-derived M1 or M2 macrophage exosomes	Preferential accumulation in cancer cells; remarkable effect of cisplatin-resistant M1 macrophage on tumor killing	196
Light-induced carbon monoxide delivery systems		Exogenous carbon monoxide	Increase PARP cleavage; reduce GSH and nuclear MT expression in cells	197
Folate-drug conjugates		Hyaluronic acid- and folic acid-based hydrogel	Inhibit the migration of ovarian cancer cells; modulate the proliferation and the expression of epithelial-mesenchymal transition-related proteins	198
		EC145	Load FR maximally in the tumor while avoiding prolonged marrow exposure; lack of hematologic toxicity	199

### Platinum drugs and other chemotherapy drugs

Cisplatin, due to resistance and side effects such as nephrotoxicity and neurotoxicity, has little chance of working better as the first-generation platinum anti-cancer drug. Many cisplatin analogues have been developed, with the hope of using them as alternatives to improve cisplatin resistance in ovarian cancer. In common with cisplatin, carboplatin is effective for patients with advanced ovarian cancer with low toxicity.^126^^,^^127^ However, partial cross-resistance between carboplatin and cisplatin limits its application in the treatment of cisplatin-resistant ovarian cancer.^128^ Most third-generation platinum chemotherapeutics, including oxaliplatin and lobaplatin, have no cross-resistance with cisplatin, from which cisplatin-resistant patients can benefit.^129^

Microtubule stabilizing agents induce mitotic arrest and lead to cell death. Taxanes are already one of the most successful and widely used natural anti-cancer drugs.^130^ The combination of cisplatin and paclitaxel still serves as first-line therapy for advanced ovarian cancer.^131^^,^^132^ However, the more effective anti-tumor effects of paclitaxel are accompanied by strong toxicity, which can cause a series of adverse effects, such as body aches, limb numbness, and hair loss.^133^ The substitution of docetaxel for paclitaxel in this regimen is also under investigation. Docetaxel is an improved drug based on paclitaxel that inhibits the formation of invasion and migration activities by affecting microtubule remodeling and membrane protein aggregation in SKOV-3 cells.^134^

Topotecan prevents DNA replication in cancer cells by inhibiting topoisomerase I.^135^ Numerous studies have explored newly administered formulations with different doses and therapy treatments to find the optimal topotecan regimen in the treatment of ovarian cancer.^136^ Pegylated liposomal doxorubicin is a formulation of liposomal doxorubicin wrapped in polyethylene glycol, which reduces cardiotoxicity while maintaining good anti-tumor effects and increasing its circulating half-life.^137^ Gemcitabine is a synthetic cytosine nucleoside derivative used in chemotherapy, which exhibits strong radio-sensitization and weak toxicity.^138^ In addition, topotecan, pegylated liposomal doxorubicin, gemcitabine, and pemetrexed are commonly used chemotherapeutic drugs in the treatment of cisplatin resistance. These drugs cause the arrest of DNA replication and induction of apoptosis, thus inhibiting ovarian cancer tumor growth, and have shown better prognostic effects in clinical experiments.^136^^,^^139^, ^140^, ^141^, ^142^, ^143^

## Targeted therapy

### Anti-angiogenic agents

Tumor growth is closely related to angiogenesis, in which vascular endothelial growth factor plays a dominant role.^144^ Humanized monoclonal anti-vascular endothelial growth factor antibodies, such as bevacizumab, have been used in the treatment of ovarian cancer. A phase II trial evaluated the efficacy of bevacizumab monotherapy in platinum-resistant epithelial ovarian cancer. The median progression-free survival of patients was 4.4 months (95% confidence interval [CI], 3.1–5.5), which confirmed that bevacizumab has single-agent activity in platinum-resistant patients.^145^ Bevacizumab combined with chemotherapy is more effective than chemotherapy alone.^146^^,^^147^ The progression-free survival of platinum-resistant ovarian cancer patients who received treatment of bevacizumab plus chemotherapy was 4 months (95% CI, 3.0–5.7), compared with 3.1 months (95% CI, 2.5–4.6) for the other group that received chemotherapy only.^148^ A phase II study of ramucirumab, a fully human anti-vascular endothelial growth factor receptor 2 antibody, showed that the median progression-free survival in the 45 cisplatin-resistant patients was 3.2 months (95% CI, 1.8–3.7) and median overall survival was 10.0 months (95% CI, 7.7–15.2).^149^ Apatinib, which specifically binds to and inhibits vascular endothelial growth factor receptor 2, is a tyrosine kinase inhibitor used orally.^150^ Twenty-eight patients with platinum-resistant recurrent epithelial ovarian cancer were selected to conduct a real-world study on the effectiveness of apatinib monotherapy.^151^ The median progression-free survival was 5.0 months and overall survival was 9.5 months, indicating that the efficacy of apatinib may be better than bevacizumab. After the combination of apatinib and etoposide, the median progression-free survival was extended to 6.0 months, and the longest progression-free survival was continued to 20.0 months, which were significantly longer than those of monotherapy treatment.^152^

### PARP inhibitors

PARP inhibitors are clinically successful agents that induce synthetic lethality by impacting DNA repair pathways to cause apoptosis and enhanced sensitivity of chemotherapy drugs.^153^ A study of breast cancer early onset gene 1/2-mutated ovarian cancer patients showed that the antitumor activity of olaparib was associated with platinum sensitivity.^154^ A GEICO phase II trial reported therapeutic effects for the combination of olaparib and pegylated liposomal doxorubicin in platinum-resistant ovarian cancer.^155^ The objective response rate in their trial was 29%, which is higher than that in platinum-resistant ovarian cancer patients treated with olaparib monotherapy.^156^ Several PARP inhibitors, such as rucaparib and niraparib, have also been approved for ovarian cancer treatment.^157^^,^^158^ Currently, under investigation in early-phase trials, talazoparib and veliparib are promising PARP inhibitors.^159^

However, patients will eventually develop resistance to PARP inhibitors. Researchers found that through mutations and up-regulation of breast cancer early onset genes, the homologous recombination repair way was likely recovered, which could lead to resistance to PARP inhibitors.^160^ There was a striking finding and highly promising for patients as resistance to PARP inhibitors and platinum in ovarian cancer could be reversed by combining PARP with ataxia-telangiectasia-mutated-and-Rad3-related kinase inhibition.^161^

### Cell-cycle kinases inhibitors

Cell-cycle kinase inhibitors, such as cyclin-dependent kinase 4/6 inhibitors, can block tumor growth by inhibiting cell-cycle progression.^162^ Both inhibitors, including ribociclib, palbociclib, and abemaciclib, are currently the only class of cyclin-dependent kinase inhibitors approved for clinical use.^163^, ^164^, ^165^ Palbociclib was the first cyclin-dependent kinase 4/6 inhibitor developed and tested in a clinical trial.^164^ Palbociclib showed longer progression-free survival and excellent tolerance, revealing the potential therapeutic effects of cyclin-dependent kinase 4/6 inhibitors in recurrent ovarian cancer.^166^ The novel compound ZC-22, a PARP and cyclin-dependent kinase 4/6 dual inhibitor, shows high therapeutic potential to significantly improve the response of ovarian cancer to cisplatin. It inhibits ovarian cancer cells by inducing cell cycle arrest and DNA damage *in vitro* and *in vivo*.^167^ Down-regulation of the expression of cyclin-dependent kinase 5 (involved in DNA repair) has been correlated with the sensitivity of A2780CIS cells to cisplatin.^168^ Dinaciclib can inhibit the expression of cyclin-dependent kinase 5 in cisplatin-resistant cell lines. The combination of dinacoxib and cisplatin for ovarian cancer treatment may provide clinical benefits.^169^

### Immunotherapy

Immunotherapy has developed rapidly over the past decade as a new direction to overcome cisplatin resistance in ovarian cancer. It controls and kills tumor cells by mobilizing the immune system.^170^ The main immune checkpoint inhibitors currently under investigation include cytotoxic T lymphocyte antigen 4, programmed cell death protein 1 (PD-1), and programmed cell death 1 ligand 1 (PD-L1) inhibitors.^171^ The latter two inhibitors have been widely studied in drug resistance treatment of ovarian cancer, while the former is seldom.

Hamanishi et al^172^ revealed the merits of nivolumab, a PD-1 inhibitor, in cisplatin-resistant ovarian cancer, showing that the median progression-free survival was 3.5 months (95% CI, 1.7–3.9 months) and median overall survival was 20.0 months. In addition, the use of nivolumab in platinum-resistant patients results in partial response to other chemotherapeutic agents, such as pegylated liposomal doxorubicin and nedaplatin, as subsequent treatment.^173^^,^^174^ Pembrolizumab, an antibody specific for PD-1, prevents PD-1 ligation by both PD-L1 and PD-L2.^175^, ^176^, ^177^ Pembrolizumab combined with niraparib appeared to be well tolerated in platinum-sensitive ovarian cancer, providing new treatment options for patients.^177^ PD-L1 is a ligand for the PD-1 receptor. The combination of a PD-L1 inhibitor with a PD-1 inhibitor may cause apoptosis in T cells, affecting immune function.^178^ One study investigated 124 patients to determine whether the combination of the anti-PD-L1 inhibitor atezolizumab and bevacizumab was synergistic in cisplatin-resistant ovarian cancer.^179^ Proliferation, migration, and invasion of A2780CIS cells were synergistically inhibited *in vitro.* In addition, other immunosuppressants in combination with a variety of drugs have exerted sustained effects on cisplatin-resistant ovarian cancer patients.^180^, ^181^, ^182^, ^183^, ^184^

### Drug delivery methods

Due to side effects, inactivation of anti-cancer drugs, and tumor heterogeneity, it is difficult to completely solve the problem of cisplatin resistance with drug treatment only. Improvement of drug delivery may produce surprising results in overcoming resistance. Drug delivery systems enable accurate drug delivery and directed release.^185^

### Nanoparticle delivery systems

Nanocarrier-based anti-cancer drug delivery systems have been intensively studied and developed. Compared with conventional anti-cancer drugs, nanocarriers have good biocompatibility and high bioavailability, which protect drugs from decomposition and promote targeting drugs to the tumor site. At present, a variety of nanocarriers, including lipids, polymers, and inorganic molecules, have been developed.^186^

Liposomes consist of spherical lipid bilayers containing water centers that can be loaded with both hydrophilic and hydrophobic drugs.^187^ Carboplatin and paclitaxel are encapsulated in cross-linked multilamellar liposome vesicles and delivered to ovarian cancer cells to reduce systemic cytotoxicity and produce stronger anti-tumor effects.^188^

Similar to lipids, polymers can increase the intracellular accumulation of therapeutic agents and enhance therapeutic efficacy through conjugation-targeting parts.^189^ Delivery of poly (lactic-co-glycolic acid)-poly (ethylene glycol) (PLGA-PEG) nanoparticles loaded with cisplatin and a DNA repair inhibitor, wortmannin, significantly reduced tumor burden, enhanced chemotherapeutic effects, and reversed cisplatin resistance.^190^ The use of polymeric nanoparticle methoxy poly (ethylene glycol)-poly (l-lactic acid) (MPEG-PLA) packaging of doxorubicin and the multi-drugs resistant protein inhibitor verapamil, was able to avoid the systemic toxicity and effectively reverse drug resistance in ovarian cancer.^191^

Inorganic nanoparticles include gold nanoparticles, silica nanoparticles, and iron oxide nanoparticles.^192^, ^193^, ^194^ A new type of anti-tumor nanoparticles, virus-like nanoparticles, are protein nano-empty shell structures without viral nucleic acid.^195^ These nanoparticles help to increase drug cytotoxicity, reverse cisplatin resistance, and achieve good effects for ovarian cancer treatment. Many nanoparticles are under investigation in clinical trials for targeted therapy in ovarian cancer, which has practical significance for reversing cisplatin resistance in ovarian cancer.

### Exosomes

Exosome carriers combine the advantages of cellular drug delivery and nanotechnology. A study assessed the feasibility of cisplatin in the treatment of ovarian cancer by exosomes and confirmed exosomes were a potentially new tool for drug delivery.^196^ Incorporation of cisplatin into umbilical cord blood-derived M1 macrophage exosomes caused a 3.3-fold increased cytotoxicity in A2780CIS cells compared with chemotherapy alone.

### Light-induced carbon monoxide delivery systems

Recently, there has been innovative research on cisplatin resistance in ovarian cancer using a light-induced carbon monoxide delivery method.^197^ Strong evidence suggests that exogenous carbon monoxide mediates inactivation of cystathionine β-synthase. Cystathionine β-synthase lowers the levels of GSH and metallothionein and prevents hindering the interaction between cisplatin and DNA, thus conferring sensitivity to cisplatin-resistant cells.

### Folate-drug conjugates

Folic acid is a water-soluble vitamin that plays a crucial role in protein synthesis, cell division, and growth in tumor cells. Due to the overexpression of folate receptor α in ovarian cancer patients, the macro-molecular coupling drugs formed by coupling the targeting ligand folic acid and anti-tumor drugs have shown promise. Serini et al^198^ constructed a cisplatin delivery hydrogel based on hyaluronic acid and folic acid, which effectively inhibited the migration of A2780CIS cells and significantly reduced epithelial-mesenchymal transition. Vintafolide (EC145), a folic acid-des-acetyl-vinblastine conjugate, shows excellent survival advantage, thus showing promise for folate-receptor-targeted therapy.^199^

## Conclusions and future perspectives

As the most widely used drug in various cancer chemotherapy, cisplatin brings substantial benefits to patients. It is the first-line drug used in the treatment of ovarian cancer. During long-term chemotherapy, most patients have a lower effective response and eventually suffer from severe cisplatin resistance. Cisplatin resistance in ovarian cancer is a difficult treatment point that affects the prognosis of patients.

There are many mechanisms of cisplatin resistance, and one or several changes may lead to drug resistance. Many molecular mechanisms have been linked to drug resistance in ovarian cancer, such as drug metabolism, autophagy, and DNA damage repair. However, the mechanism underlying cisplatin resistance in ovarian cancer has not been fully elucidated. For example, the ROS regulation mentioned in this review has opposite effects on cisplatin resistance. In addition, the dynamic evolution of tumor heterogeneity produces resistant subsets, which increase the difficulties of cancer treatment. The biological relationships between different subpopulations in tumors and between subpopulations and the microenvironment are also unclear. A clear definition of the molecular mechanism underlying cisplatin resistance will greatly aid in the development of treatments to address drug resistance in ovarian cancer.

Many drugs, including non-platinum chemotherapeutic drugs, anti-angiogenic agents, immunosuppressants, and PARP inhibitors, have been shown to sensitize cisplatin-resistant ovarian cells. Although some drugs have been investigated in clinical trials, many have not achieved satisfactory clinical results. A considerable number of the studies have been terminated due to serious adverse reactions, which suggests that researchers should pay attention to the adverse effects of drugs in the process of exploring targeted therapy. Multi-drug combination therapy for cisplatin-resistant ovarian cancer seems to be a good therapeutic strategy to overcome cisplatin resistance. It is hoped that multiple drugs will work synergistically. However, the determination of combinations of drugs and appropriate loading methods, which can be determined based on drug metabolism and the pharmacokinetic diversity of different drugs, has also been a great challenge in the treatment of cisplatin-resistant ovarian cancer. The cost and difficulties of making the preparations also need to be considered. Overall, this review summarized the current mechanisms and therapeutic strategies of cisplatin resistance in ovarian cancer, which could pave the way for the development of resistance therapy.

## Author contributions

Zhihui Zhu planned the manuscript. Chenying Jiang wrote the first draft, with substantial inputs and comments from Zhihui Zhu, Chenjun Shen, Maowei Ni, and other authors. All authors read and approved the final version.

## Conflict of interests

The authors declare that there is no conflict of interests.

## Funding

This work was supported by the 10.13039/501100001809National Natural Science Foundation of China (No. 82104448) and the Key Research Project of Traditional Chinese Medicine in Zhejiang Province, China (No. 2022ZZ008).

## References

[1] Doherty J.A., Peres L.C., Wang C., Way G.P., Greene C.S., Schildkraut J.M. (2017). Challenges and opportunities in studying the epidemiology of ovarian cancer subtypes. Curr Epidemiol Rep.

[2] Armstrong D.K., Alvarez R.D., Backes F.J. (2022). NCCN guidelines® insights: ovarian cancer, version 3.2022. J Natl Compr Cancer Netw.

[3] Lheureux S., Gourley C., Vergote I., Oza A.M. (2019). Epithelial ovarian cancer. Lancet.

[4] Siegel R.L., Miller K.D., Wagle N.S., Jemal A. (2023). Cancer statistics, 2023. CA A Cancer J Clin.

[5] Torre L.A., Trabert B., DeSantis C.E. (2018). Ovarian cancer statistics, 2018. CA A Cancer J Clin.

[6] Kurnit K.C., Fleming G.F., Lengyel E. (2021). Updates and new options in advanced epithelial ovarian cancer treatment. Obstet Gynecol.

[7] Webb P.M., Jordan S.J. (2017). Epidemiology of epithelial ovarian cancer. Best Pract Res Clin Obstet Gynaecol.

[8] Ghosh S. (2019). Cisplatin: the first metal based anticancer drug. Bioorg Chem.

[9] Florea A.M., Büsselberg D. (2011). Cisplatin as an anti-tumor drug: cellular mechanisms of activity, drug resistance and induced side effects. Cancers.

[10] Dasari S., Tchounwou P.B. (2014). Cisplatin in cancer therapy: molecular mechanisms of action. Eur J Pharmacol.

[11] Yang L., Xie H.J., Li Y.Y., Wang X., Liu X.X., Mai J. (2022). Molecular mechanisms of platinum-based chemotherapy resistance in ovarian cancer. Oncol Rep.

[12] Buechel M., Herzog T.J., Westin S.N., Coleman R.L., Monk B.J., Moore K.N. (2019). Treatment of patients with recurrent epithelial ovarian cancer for whom platinum is still an option. Ann Oncol.

[13] Sarwar S., Alamro A.A., Alghamdi A.A. (2021). Enhanced accumulation of cisplatin in ovarian cancer cells from combination with wedelolactone and resulting inhibition of multiple epigenetic drivers. Drug Des Dev Ther.

[14] Sarkhosh-Inanlou R., Molaparast M., Mohammadzadeh A., Shafiei-Irannejad V. (2020). Sanguinarine enhances cisplatin sensitivity via glutathione depletion in cisplatin-resistant ovarian cancer (A2780) cells. Chem Biol Drug Des.

[15] Xiao Y., Lin F.T., Lin W.C. (2021). ACTL6A promotes repair of cisplatin-induced DNA damage, a new mechanism of platinum resistance in cancer. Proc Natl Acad Sci U S A.

[16] Cocetta V., Ragazzi E., Montopoli M. (2020). Links between cancer metabolism and cisplatin resistance. Int Rev Cell Mol Biol.

[17] Tchounwou P.B., Dasari S., Noubissi F.K., Ray P., Kumar S. (2021). Advances in our understanding of the molecular mechanisms of action of cisplatin in cancer therapy. J Exp Pharmacol.

[18] Keshtkar S., Azarpira N., Ghahremani M.H. (2018). Mesenchymal stem cell-derived extracellular vesicles: novel frontiers in regenerative medicine. Stem Cell Res Ther.

[19] Wang X., Jiang L., Liu Q. (2022). miR-18a-5p derived from mesenchymal stem cells-extracellular vesicles inhibits ovarian cancer cell proliferation, migration, invasion, and chemotherapy resistance. J Transl Med.

[20] Luo Y., Gui R. (2020). Circulating exosomal circFoxp1 confers cisplatin resistance in epithelial ovarian cancer cells. J Gynecol Oncol.

[21] Carey P., Low E., Harper E., Stack M.S. (2021). Metalloproteinases in ovarian cancer. Int J Mol Sci.

[22] Wang S., Jia J., Liu D. (2019). Matrix metalloproteinase expressions play important role in prediction of ovarian cancer outcome. Sci Rep.

[23] Laios A., Mohamed B.M., Kelly L. (2013). Pre-Treatment of platinum resistant ovarian cancer cells with an MMP-9/MMP-2 inhibitor prior to cisplatin enhances cytotoxicity as determined by high content screening. Int J Mol Sci.

[24] Pietilä E.A., Gonzalez-Molina J., Moyano-Galceran L. (2021). Co-evolution of matrisome and adaptive adhesion dynamics drives ovarian cancer chemoresistance. Nat Commun.

[25] Sherman-Baust C.A., Weeraratna A.T., Rangel L.B. (2003). Remodeling of the extracellular matrix through overexpression of collagen VI contributes to cisplatin resistance in ovarian cancer cells. Cancer Cell.

[26] Arnesano F., Natile G. (2021). Interference between copper transport systems and platinum drugs. Semin Cancer Biol.

[27] Pan H., Kim E., Rankin G.O., Rojanasakul Y., Tu Y., Chen Y.C. (2018). Theaflavin-3, 3'-digallate enhances the inhibitory effect of cisplatin by regulating the copper transporter 1 and glutathione in human ovarian cancer cells. Int J Mol Sci.

[28] Januchowski R., Zawierucha P., Ruciński M. (2014). Drug transporter expression profiling in chemoresistant variants of the A2780 ovarian cancer cell line. Biomed Pharmacother.

[29] Lancaster C.S., Sprowl J.A., Walker A.L., Hu S., Gibson A.A., Sparreboom A. (2013). Modulation of OATP1B-type transporter function alters cellular uptake and disposition of platinum chemotherapeutics. Mol Cancer Therapeut.

[30] Freitas-Lima L.C., Budu A., Arruda A.C. (2020). PPAR-α deletion attenuates cisplatin nephrotoxicity by modulating renal organic transporters MATE-1 and OCT-2. Int J Mol Sci.

[31] Spreckelmeyer S., van der Zee M., Bertrand B., Bodio E., Stürup S., Casini A. (2018). Relevance of copper and organic cation transporters in the activity and transport mechanisms of an anticancer cyclometallated gold(III) compound in comparison to cisplatin. Front Chem.

[32] Mariniello M., Petruzzelli R., Wanderlingh L.G. (2020). Synthetic lethality screening identifies FDA-approved drugs that overcome ATP7B-mediated tolerance of tumor cells to cisplatin. Cancers.

[33] Lukanović D., Herzog M., Kobal B., Černe K. (2020). The contribution of copper efflux transporters ATP7A and ATP7B to chemoresistance and personalized medicine in ovarian cancer. Biomed Pharmacother.

[34] Wang Y., Liu M., Zhang J. (2018). Multidrug resistance protein 1 deficiency promotes doxorubicin-induced ovarian toxicity in female mice. Toxicol Sci.

[35] Zheng X., Andruska N., Lambrecht M.J. (2018). Targeting multidrug-resistant ovarian cancer through estrogen receptor α dependent ATP depletion caused by hyperactivation of the unfolded protein response. Oncotarget.

[36] Nunes S.C., Serpa J. (2018). Glutathione in ovarian cancer: a double-edged sword. Int J Mol Sci.

[37] Guo J., Satoh K., Tabata S., Mori M., Tomita M., Soga T. (2021). Reprogramming of glutamine metabolism via glutamine synthetase silencing induces cisplatin resistance in A2780 ovarian cancer cells. BMC Cancer.

[38] Tao W., He Z. (2018). ROS-responsive drug delivery systems for biomedical applications. Asian J Pharm Sci.

[39] Kleih M., Böpple K., Dong M. (2019). Direct impact of cisplatin on mitochondria induces ROS production that dictates cell fate of ovarian cancer cells. Cell Death Dis.

[40] Inoue M., Sato E.F., Nishikawa M. (2003). Mitochondrial generation of reactive oxygen species and its role in aerobic life. Curr Med Chem.

[41] Lv B., Ma J., Wang Y., Qu X., Qiu J., Hua K. (2022). Mitochondria-targeted mesoporous organic silica nanoplatforms for overcoming cisplatin resistance by disturbing mitochondrial redox homeostasis. Front Chem.

[42] Han Y., Kim B., Cho U. (2019). Mitochondrial fission causes cisplatin resistance under hypoxic conditions via ROS in ovarian cancer cells. Oncogene.

[43] Cui Q., Wang J.Q., Assaraf Y.G. (2018). Modulating ROS to overcome multidrug resistance in cancer. Drug Resist Updates.

[44] Onorati A.V., Dyczynski M., Ojha R., Amaravadi R.K. (2018). Targeting autophagy in cancer. Cancer.

[45] Filomeni G., De Zio D., Cecconi F. (2015). Oxidative stress and autophagy: the clash between damage and metabolic needs. Cell Death Differ.

[46] Li X., Hu X., Wang J. (2018). Inhibition of autophagy via activation of the PI3K/Akt/mTOR pathway contributes to the protection of hesperidin against myocardial ischemia/reperfusion injury. Int J Mol Med.

[47] Jiang S., Chang H., Deng S., Fan D. (2019). Icariin enhances the chemosensitivity of cisplatin-resistant ovarian cancer cells by suppressing autophagy via activation of the AKT/mTOR/ATG5 pathway. Int J Oncol.

[48] Mou Y., Wang J., Wu J. (2019). Ferroptosis, a new form of cell death: opportunities and challenges in cancer. J Hematol Oncol.

[49] Xuan Y., Wang H., Yung M.M. (2022). SCD1/FADS2 fatty acid desaturases equipoise lipid metabolic activity and redox-driven ferroptosis in ascites-derived ovarian cancer cells. Theranostics.

[50] Ni M., Zhou J., Zhu Z. (2023). Shikonin and cisplatin synergistically overcome cisplatin resistance to ovarian cancer by inducing ferroptosis via upregulation of HMOX1 to promote Fe^2+^ accumulation. Phytomedicine.

[51] Cheng Q., Bao L., Li M., Chang K., Yi X. (2021). Erastin synergizes with cisplatin via ferroptosis to inhibit ovarian cancer growth *in vitro* and *in vivo*. J Obstet Gynaecol Res.

[52] Abbaszadeh Z., Çeşmeli S., Biray Avcı Ç. (2020). Crucial players in glycolysis: cancer progress. Gene.

[53] Cao Y. (2019). Adipocyte and lipid metabolism in cancer drug resistance. J Clin Invest.

[54] Li M., Chen X., Wang X. (2021). RSL3 enhances the antitumor effect of cisplatin on prostate cancer cells via causing glycolysis dysfunction. Biochem Pharmacol.

[55] Hua G., Zeng Z.L., Shi Y.T., Chen W., He L.F., Zhao G.F. (2021). LncRNA XIST contributes to cisplatin resistance of lung cancer cells by promoting cellular glycolysis through sponging miR-101-3p. Pharmacology.

[56] Xintaropoulou C., Ward C., Wise A. (2018). Expression of glycolytic enzymes in ovarian cancers and evaluation of the glycolytic pathway as a strategy for ovarian cancer treatment. BMC Cancer.

[57] Zhang X.Y., Zhang M., Cong Q. (2018). Hexokinase 2 confers resistance to cisplatin in ovarian cancer cells by enhancing cisplatin-induced autophagy. Int J Biochem Cell Biol.

[58] Yang X., Li Z., Ren H., Peng X., Fu J. (2022). New progress of glutamine metabolism in the occurrence, development, and treatment of ovarian cancer from mechanism to clinic. Front Oncol.

[59] Yang W.H., Qiu Y., Stamatatos O., Janowitz T., Lukey M.J. (2021). Enhancing the efficacy of glutamine metabolism inhibitors in cancer therapy. Trends Cancer.

[60] Obrist F., Michels J., Durand S. (2018). Metabolic vulnerability of cisplatin-resistant cancers. EMBO J.

[61] Fasoulakis Z., Koutras A., Ntounis T. (2023). Ovarian cancer and glutamine metabolism. Int J Mol Sci.

[62] Si M., Lang J. (2018). The roles of metallothioneins in carcinogenesis. J Hematol Oncol.

[63] Merlos Rodrigo M.A., Jimenez Jimemez A.M., Haddad Y. (2020). Metallothionein isoforms as double agents - their roles in carcinogenesis, cancer progression and chemoresistance. Drug Resist Updates.

[64] Surowiak P., Materna V., Maciejczyk A. (2007). Nuclear metallothionein expression correlates with cisplatin resistance of ovarian cancer cells and poor clinical outcome. Virchows Arch.

[65] Rocha C.R.R., Silva M.M., Quinet A., Cabral-Neto J.B., Menck C.F.M. (2018). DNA repair pathways and cisplatin resistance: an intimate relationship. Clinics.

[66] Krasikova Y., Rechkunova N., Lavrik O. (2021). Nucleotide excision repair: from molecular defects to neurological abnormalities. Int J Mol Sci.

[67] Ceccaldi R., O'Connor K.W., Mouw K.W. (2015). A unique subset of epithelial ovarian cancers with platinum sensitivity and PARP inhibitor resistance. Cancer Res.

[68] Zhao M., Li S., Zhou L., Shen Q., Zhu H., Zhu X. (2018). Prognostic values of excision repair cross-complementing genes mRNA expression in ovarian cancer patients. Life Sci.

[69] Du P., Zhang X., Liu H., Chen L. (2015). Lentivirus-Mediated RNAi silencing targeting ERCC1 reverses cisplatin resistance in cisplatin-resistant ovarian carcinoma cell line. DNA Cell Biol.

[70] Mesquita K.A., Alabdullah M., Griffin M. (2019). ERCC1-XPF deficiency is a predictor of olaparib induced synthetic lethality and platinum sensitivity in epithelial ovarian cancers. Gynecol Oncol.

[71] Cierna Z., Miskovska V., Roska J. (2020). Increased levels of XPA might be the basis of cisplatin resistance in germ cell tumours. BMC Cancer.

[72] Lin K., Ye D., Xie X. (2008). Protein expression levels of excision repair cross-complementation group 1 and xeroderma pigmentosum D correlate with response to platinum-based chemotherapy in the patients with advanced epithelial ovarian cancer. Int J Gynecol Cancer.

[73] Zebian A., Shaito A., Mazurier F., Rezvani H.R., Zibara K. (2019). XPC beyond nucleotide excision repair and skin cancers. Mutat Res Rev Mutat Res.

[74] Fleming N.D., Agadjanian H., Nassanian H. (2012). Xeroderma pigmentosum complementation group C single-nucleotide polymorphisms in the nucleotide excision repair pathway correlate with prolonged progression-free survival in advanced ovarian cancer. Cancer.

[75] Vergote I., González-Martín A., Ray-Coquard I. (2022). European experts consensus: BRCA/homologous recombination deficiency testing in first-line ovarian cancer. Ann Oncol.

[76] Ouyang J., Yadav T., Zhang J.M. (2021). RNA transcripts stimulate homologous recombination by forming DR-loops. Nature.

[77] Daley J.M., Gaines W.A., Kwon Y., Sung P. (2014). Regulation of DNA pairing in homologous recombination. Cold Spring Harbor Perspect Biol.

[78] Alsop K., Fereday S., Meldrum C. (2012). BRCA mutation frequency and patterns of treatment response in BRCA mutation-positive women with ovarian cancer: a report from the Australian Ovarian Cancer Study Group. J Clin Oncol.

[79] Bartz S.R., Zhang Z., Burchard J. (2006). Small interfering RNA screens reveal enhanced cisplatin cytotoxicity in tumor cells having both BRCA network and TP53 disruptions. Mol Cell Biol.

[80] Feng Y., Wang D., Xiong L., Zhen G., Tan J. (2021). Predictive value of RAD51 on the survival and drug responsiveness of ovarian cancer. Cancer Cell Int.

[81] Hua T., Li Y., Li X.F., Sun H.Y., Chen J., Kang S. (2019). Hypermethylation of mismatch repair gene hMSH2 associates with platinum-resistant disease in epithelial ovarian cancer. Clin Epigenet.

[82] Prakash R., Zhang Y., Feng W., Jasin M. (2015). Homologous recombination and human health: the roles of BRCA1, BRCA2, and associated proteins. Cold Spring Harbor Perspect Biol.

[83] Boeckman H.J., Trego K.S., Turchi J.J. (2005). Cisplatin sensitizes cancer cells to ionizing radiation via inhibition of nonhomologous end joining. Mol Cancer Res.

[84] Liu Y., Xu Y., Jiang M., Chen W., Zhu X. (2021). Significant value of XRCC2 and XRCC9 expression in the prognosis of human ovarian carcinoma. J Cancer.

[85] Beermann J., Piccoli M.T., Viereck J., Thum T. (2016). Non-coding RNAs in development and disease: background, mechanisms, and therapeutic approaches. Physiol Rev.

[86] Taheri M., Shoorei H., Tondro Anamag F., Ghafouri-Fard S., Dinger M.E. (2021). LncRNAs and miRNAs participate in determination of sensitivity of cancer cells to cisplatin. Exp Mol Pathol.

[87] Lu T.X., Rothenberg M.E. (2018). microRNA. J Allergy Clin Immunol.

[88] Yu P.N., Yan M.D., Lai H.C. (2014). Downregulation of miR-29 contributes to cisplatin resistance of ovarian cancer cells. Int J Cancer.

[89] Liu Y., Niu Z., Lin X., Tian Y. (2017). miR-216b increases cisplatin sensitivity in ovarian cancer cells by targeting PARP1. Cancer Gene Ther.

[90] Xu Z.H., Yao T.Z., Liu W. (2018). miR-378a-3p sensitizes ovarian cancer cells to cisplatin through targeting MAPK1/GRB2. Biomed Pharmacother.

[91] Zhang X.A., Huang L., Zhao Y., Tan W. (2013). Downregulation of miR-130a contributes to cisplatin resistance in ovarian cancer cells by targeting X-linked inhibitor of apoptosis (XIAP) directly. Acta Biochim Biophys Sin.

[92] Yan M., Yang X., Shen R. (2018). miR-146b promotes cell proliferation and increases chemosensitivity, but attenuates cell migration and invasion via FBXL10 in ovarian cancer. Cell Death Dis.

[93] Chen S., Jiao J.W., Sun K.X., Zong Z.H., Zhao Y. (2015). microRNA-133b targets glutathione S-transferase π expression to increase ovarian cancer cell sensitivity to chemotherapy drugs. Drug Des Dev Ther.

[94] Sun K.X., Jiao J.W., Chen S., Liu B.L., Zhao Y. (2015). microRNA-186 induces sensitivity of ovarian cancer cells to paclitaxel and cisplatin by targeting ABCB1. J Ovarian Res.

[95] Zhang X.L., Sun B.L., Tian S.X., Li L., Zhao Y.C., Shi P.P. (2019). microRNA-132 reverses cisplatin resistance and metastasis in ovarian cancer by the targeted regulation on Bmi-1. Eur Rev Med Pharmacol Sci.

[96] Ge T., Liu T., Guo L., Chen Z., Lou G. (2020). microRNA-302 represses epithelial-mesenchymal transition and cisplatin resistance by regulating ATAD2 in ovarian carcinoma. Exp Cell Res.

[97] Xiang Y., Ma N., Wang D. (2014). miR-152 and miR-185 co-contribute to ovarian cancer cells cisplatin sensitivity by targeting DNMT1 directly: a novel epigenetic therapy independent of decitabine. Oncogene.

[98] Wang J., Liu L. (2021). miR-149-3p promotes the cisplatin resistance and EMT in ovarian cancer through downregulating TIMP2 and CDKN1A. J Ovarian Res.

[99] Chen L., Wang J., Liu Q. (2022). Long noncoding RNAs as therapeutic targets to overcome chemoresistance in ovarian cancer. Front Cell Dev Biol.

[100] Zhu Y., Yang L., Wang J., Li Y., Chen Y. (2022). SP1-induced lncRNA MCF2L-AS1 promotes cisplatin resistance in ovarian cancer by regulating IGF_2_BP_1_/IGF2/MEK/ERK axis. J Gynecol Oncol.

[101] Lin C., Zheng M., Yang Y. (2022). Knockdown of lncRNA ACTA2-AS1 reverses cisplatin resistance of ovarian cancer cells via inhibition of miR-378a-3p-regulated Wnt5a. Bioengineered.

[102] Noriega-Rivera R., Rivera-Serrano M., Rabelo-Fernandez R.J., Pérez-Santiago J., Valiyeva F., Vivas-Mejía P.E. (2022). Upregulation of the long noncoding RNA CASC10 promotes cisplatin resistance in high-grade serous ovarian cancer. Int J Mol Sci.

[103] Chen Y., Cui Z., Wu Q., Wang H., Xia H., Sun Y. (2022). Long non-coding RNA HOXA11-AS knockout inhibits proliferation and overcomes drug resistance in ovarian cancer. Bioengineered.

[104] Wu Y., Wang T., Xia L., Zhang M. (2021). LncRNA WDFY3-AS2 promotes cisplatin resistance and the cancer stem cell in ovarian cancer by regulating hsa-miR-139-5p/SDC4 axis. Cancer Cell Int.

[105] Li X., Zhang Y., Wang X. (2022). Long non-coding RNA CTSLP8 mediates ovarian cancer progression and chemotherapy resistance by modulating cellular glycolysis and regulating c-Myc expression through PKM2. Cell Biol Toxicol.

[106] Xiao L., Shi X.Y., Li Z.L. (2021). Downregulation of LINC01508 contributes to cisplatin resistance in ovarian cancer via the regulation of the Hippo-YAP pathway. J Gynecol Oncol.

[107] Elsayed A.M., Bayraktar E., Amero P. (2021). PRKAR1B-AS2 long noncoding RNA promotes tumorigenesis, survival, and chemoresistance via the PI3K/AKT/mTOR pathway. Int J Mol Sci.

[108] Xu R., Peng H., Yang N., Liu Z., Lu W. (2023). Nuclear lncRNA CERNA1 enhances the cisplatin-induced cell apoptosis and overcomes chemoresistance via epigenetic activation of BCL2L10 in ovarian cancer. Genes Dis.

[109] Huang K., Chen X., Geng Z. (2023). LncRNA SLC25A21-AS1 increases the chemosensitivity and inhibits the progression of ovarian cancer by upregulating the expression of KCNK_4_. Funct Integr Genomics.

[110] Mu Q., Lv Y., Luo C. (2021). Research progress on the functions and mechanism of circRNA in cisplatin resistance in tumors. Front Pharmacol.

[111] Shen J., Liang C., Su X. (2022). Dysfunction and ceRNA network of the tumor suppressor miR-637 in cancer development and prognosis. Biomark Res.

[112] Qin K., Zhang F., Wang H. (2023). circRNA circSnx12 confers Cisplatin chemoresistance to ovarian cancer by inhibiting ferroptosis through a miR-194-5p/SLC7A11 axis. BMB Rep.

[113] Fu L., Zhang D., Yi N. (2022). Circular RNA circPBX3 promotes cisplatin resistance of ovarian cancer cells via interacting with IGF_2_BP_2_ to stabilize ATP7A mRNA expression. Hum Cell.

[114] Rao Y., Zhang W., Li D., Li X., Ma Y., Qu P. (2022). Circ TYMP1 inhibits carcinogenesis and cisplatin resistance in ovarian cancer by reducing Smad2/3 phosphorylation via a microRNA-182A-3p/TGF1B axis. Contrast Media Mol Imaging.

[115] Zhao Z., Ji M., Wang Q., He N., Li Y. (2019). Circular RNA Cdr1as upregulates SCAI to suppress cisplatin resistance in ovarian cancer via miR-1270 suppression. Mol Ther Nucleic Acids.

[116] Liu X., Yin Z., Wu Y., Zhan Q., Huang H., Fan J. (2022). Circular RNA lysophosphatidic acid receptor 3 (circ-LPAR3) enhances the cisplatin resistance of ovarian cancer. Bioengineered.

[117] Pan G., Liu Y., Shang L., Zhou F., Yang S. (2021). EMT-associated microRNAs and their roles in cancer stemness and drug resistance. Cancer Commun.

[118] Kielbik M., Szulc-Kielbik I., Klink M. (2021). Impact of selected signaling proteins on SNAIL 1 and SNAIL 2 expression in ovarian cancer cell lines in relation to cells' cisplatin resistance and EMT markers level. Int J Mol Sci.

[119] Haslehurst A.M., Koti M., Dharsee M. (2012). EMT transcription factors snail and slug directly contribute to cisplatin resistance in ovarian cancer. BMC Cancer.

[120] Lim Z.F., Ma P.C. (2019). Emerging insights of tumor heterogeneity and drug resistance mechanisms in lung cancer targeted therapy. J Hematol Oncol.

[121] Ren W., Gao L., Song J. (2018). Structural basis of DNMT1 and DNMT3A-mediated DNA methylation. Genes.

[122] Prasetyanti P.R., Medema J.P. (2017). Intra-tumor heterogeneity from a cancer stem cell perspective. Mol Cancer.

[123] Ding J., Zhang Y., Che Y. (2022). Ovarian cancer stem cells: critical roles in anti-tumor immunity. Front Genet.

[124] Mihanfar A., Aghazadeh Attari J., Mohebbi I. (2019). Ovarian cancer stem cell: a potential therapeutic target for overcoming multidrug resistance. J Cell Physiol.

[125] Chen F., Long Q., Fu D. (2018). Targeting SPINK1 in the damaged tumour microenvironment alleviates therapeutic resistance. Nat Commun.

[126] Ozols R.F., Bundy B.N., Greer B.E. (2003). Phase III trial of carboplatin and paclitaxel compared with cisplatin and paclitaxel in patients with optimally resected stage III ovarian cancer: a Gynecologic Oncology Group study. J Clin Oncol.

[127] Falandry C., Rousseau F., Mouret-Reynier M.A. (2021). Efficacy and safety of first-line single-agent carboplatin vs carboplatin plus paclitaxel for vulnerable older adult women with ovarian cancer: a GINECO/GCIG randomized clinical trial. JAMA Oncol.

[128] Hamaguchi K., Godwin A.K., Yakushiji M., O'Dwyer P.J., Ozols R.F., Hamilton T.C. (1993). Cross-resistance to diverse drugs is associated with primary cisplatin resistance in ovarian cancer cell lines. Cancer Res.

[129] Soulié P., Bensmaïne A., Garrino C. (1997). Oxaliplatin/cisplatin (L-OHP/CDDP) combination in heavily pretreated ovarian cancer. Eur J Cancer.

[130] Weaver B.A. (2014). How Taxol/paclitaxel kills cancer cells. Mol Biol Cell.

[131] Cheng M., Lee H.H., Chang W.H. (2019). Weekly dose-dense paclitaxel and triweekly low-dose cisplatin: a well-tolerated and effective chemotherapeutic regimen for first-line treatment of advanced ovarian, fallopian tube, and primary peritoneal cancer. Int J Environ Res Publ Health.

[132] Huang C.Y., Cheng M., Lee N.R. (2020). Comparing paclitaxel-carboplatin with paclitaxel-cisplatin as the front-line chemotherapy for patients with FIGO IIIC serous-type tubo-ovarian cancer. Int J Environ Res Publ Health.

[133] Al-Mahayri Z.N., AlAhmad M.M., Ali B.R. (2021). Current opinion on the pharmacogenomics of paclitaxel-induced toxicity. Expet Opin Drug Metabol Toxicol.

[134] Hou Y., Zhao C., Xu B., Huang Y., Liu C. (2021). Effect of docetaxel on mechanical properties of ovarian cancer cells. Exp Cell Res.

[135] Lihua P., Chen X.Y., Wu T.X. (2008). Topotecan for ovarian cancer. Cochrane Database Syst Rev.

[136] Sehouli J., Stengel D., Harter P. (2011). Topotecan weekly versus conventional 5-day schedule in patients with platinum-resistant ovarian cancer: a randomized multicenter phase II trial of the north-eastern German society of gynecological oncology ovarian cancer study group. J Clin Oncol.

[137] Lawrie T.A., Bryant A., Cameron A., Gray E., Morrison J. (2013). Pegylated liposomal doxorubicin for relapsed epithelial ovarian cancer. Cochrane Database Syst Rev.

[138] Lorusso D., di Stefano A., Fanfani F., Scambia G. (2006). Role of gemcitabine in ovarian cancer treatment. Ann Oncol.

[139] Miller D.S., Blessing J.A., Krasner C.N. (2009). Phase II evaluation of pemetrexed in the treatment of recurrent or persistent platinum-resistant ovarian or primary peritoneal carcinoma: a study of the Gynecologic Oncology Group. J Clin Oncol.

[140] Sehouli J., Alvarez A.M., Manouchehrpour S. (2012). A phase II trial of pemetrexed in combination with carboplatin in patients with recurrent ovarian or primary peritoneal cancer. Gynecol Oncol.

[141] Lheureux S., Cristea M.C., Bruce J.P. (2021). Adavosertib plus gemcitabine for platinum-resistant or platinum-refractory recurrent ovarian cancer: a double-blind, randomised, placebo-controlled, phase 2 trial. Lancet.

[142] Lee M.W., Ryu H., Song I.C. (2020). Efficacy of cisplatin combined with topotecan in patients with advanced or recurrent ovarian cancer as second- or higher-line palliative chemotherapy. Medicine.

[143] McGuire W.P., Penson R.T., Gore M. (2018). Randomized phase II study of the PDGFRα antibody olaratumab plus liposomal doxorubicin versus liposomal doxorubicin alone in patients with platinum-refractory or platinum-resistant advanced ovarian cancer. BMC Cancer.

[144] Ma S., Mangala L.S., Hu W. (2021). CD63-mediated cloaking of VEGF in small extracellular vesicles contributes to anti-VEGF therapy resistance. Cell Rep.

[145] Cannistra S.A., Matulonis U.A., Penson R.T. (2007). Phase II study of bevacizumab in patients with platinum-resistant ovarian cancer or peritoneal serous cancer. J Clin Oncol.

[146] Lee J.Y., Park J.Y., Park S.Y. (2019). Real-world effectiveness of bevacizumab based on *AURELIA* in platinum-resistant recurrent ovarian cancer (REBECA): a Korean Gynecologic Oncology Group study (KGOG 3041). Gynecol Oncol.

[147] Poveda A.M., Selle F., Hilpert F. (2015). Bevacizumab combined with weekly paclitaxel, pegylated liposomal doxorubicin, or topotecan in platinum-resistant recurrent ovarian cancer: analysis by chemotherapy cohort of the randomized phase III *AURELIA* trial. J Clin Oncol.

[148] Shoji T., Enomoto T., Abe M. (2022). Efficacy and safety of standard of care with/without bevacizumab for platinum-resistant ovarian/fallopian tube/peritoneal cancer previously treated with bevacizumab: the Japanese Gynecologic Oncology Group study JGOG3023. Cancer Sci.

[149] Penson R.T., Moore K.M., Fleming G.F. (2014). A phase II study of ramucirumab (IMC-1121B) in the treatment of persistent or recurrent epithelial ovarian, fallopian tube or primary peritoneal carcinoma. Gynecol Oncol.

[150] Wang T., Tang J., Yang H. (2022). Effect of apatinib plus pegylated liposomal doxorubicin vs pegylated liposomal doxorubicin alone on platinum-resistant recurrent ovarian cancer: the APPROVE randomized clinical trial. JAMA Oncol.

[151] Zhang J., Li A., Jiang Q., Zheng F., Zhu H. (2019). Efficacy and safety of apatinib treatment in platinum-resistant recurrent epithelial ovarian cancer: a real world study. Drug Des Dev Ther.

[152] Huang Q., Chu C., Tang J., Dai Z. (2020). Efficacy and safety of apatinib combined with etoposide in patients with recurrent platinum-resistant epithelial ovarian cancer: a retrospective study. J Cancer.

[153] Franzese E., Centonze S., Diana A. (2019). PARP inhibitors in ovarian cancer. Cancer Treat Rev.

[154] Fong P.C., Yap T.A., Boss D.S. (2010). Poly(ADP)-ribose polymerase inhibition: frequent durable responses in BRCA carrier ovarian cancer correlating with platinum-free interval. J Clin Oncol.

[155] Perez-Fidalgo J.A., Cortés A., Guerra E. (2021). Olaparib in combination with pegylated liposomal doxorubicin for platinum-resistant ovarian cancer regardless of BRCA status: a GEICO phase II trial (ROLANDO study). ESMO Open.

[156] Vanderstichele A., Loverix L., Busschaert P. (2022). Randomized *CLIO*/BGOG-ov10 trial of olaparib monotherapy versus physician's choice chemotherapy in relapsed ovarian cancer. Gynecol Oncol.

[157] González-Martín A., Pothuri B., Vergote I. (2019). Niraparib in patients with newly diagnosed advanced ovarian cancer. N Engl J Med.

[158] Coleman R.L., Oza A.M., Lorusso D. (2017). Rucaparib maintenance treatment for recurrent ovarian carcinoma after response to platinum therapy (ARIEL3): a randomised, double-blind, placebo-controlled, phase 3 trial. Lancet.

[159] Mittica G., Ghisoni E., Giannone G. (2018). PARP inhibitors in ovarian cancer. Recent Pat Anti Cancer Drug Discov.

[160] McMullen M., Karakasis K., Madariaga A., Oza A.M. (2020). Overcoming platinum and PARP-inhibitor resistance in ovarian cancer. Cancers.

[161] Kim H., Xu H., George E. (2020). Combining PARP with ATR inhibition overcomes PARP inhibitor and platinum resistance in ovarian cancer models. Nat Commun.

[162] Goel S., DeCristo M.J., McAllister S.S., Zhao J.J. (2018). CDK4/6 inhibition in cancer: beyond cell cycle arrest. Trends Cell Biol.

[163] Coffman L.G., Orellana T.J., Liu T. (2022). Phase I trial of ribociclib with platinum chemotherapy in ovarian cancer. JCI Insight.

[164] Gnant M., Dueck A.C., Frantal S. (2022). Adjuvant palbociclib for early breast cancer: the PALLAS trial results (ABCSG-42/AFT-05/BIG-14-03). J Clin Oncol.

[165] Sledge G.W., Toi M., Neven P. (2020). The effect of abemaciclib plus fulvestrant on overall survival in hormone receptor-positive, ERBB2-negative breast cancer that progressed on endocrine therapy-MONARCH 2: a randomized clinical trial. JAMA Oncol.

[166] Lee D.W., Ho G.F. (2020). Palbociclib in the treatment of recurrent ovarian cancer. Gynecol Oncol Rep.

[167] Tian C., Wei Y., Li J. (2022). A novel CDK4/6 and PARP dual inhibitor ZC-22 effectively suppresses tumor growth and improves the response to cisplatin treatment in breast and ovarian cancer. Int J Mol Sci.

[168] Selvendiran K., Ahmed S., Dayton A. (2011). HO-3867, a curcumin analog, sensitizes cisplatin-resistant ovarian carcinoma, leading to therapeutic synergy through STAT3 inhibition. Cancer Biol Ther.

[169] Howard D., James D., Garcia-Parra J. (2022). Dinaciclib as an effective pan-cyclin dependent kinase inhibitor in platinum resistant ovarian cancer. Front Oncol.

[170] Lesch S., Gill S. (2021). The promise and perils of immunotherapy. Blood Adv.

[171] Gibney G.T., Weiner L.M., Atkins M.B. (2016). Predictive biomarkers for checkpoint inhibitor-based immunotherapy. Lancet Oncol.

[172] Hamanishi J., Mandai M., Ikeda T. (2015). Safety and antitumor activity of anti-PD-1 antibody, nivolumab, in patients with platinum-resistant ovarian cancer. J Clin Oncol.

[173] Inayama Y., Hamanishi J., Matsumura N. (2018). Antitumor effect of nivolumab on subsequent chemotherapy for platinum-resistant ovarian cancer. Oncol.

[174] Zamarin D., Burger R.A., Sill M.W. (2020). Randomized phase II trial of nivolumab versus nivolumab and ipilimumab for recurrent or persistent ovarian cancer: an NRG oncology study. J Clin Oncol.

[175] Liao J.B., Gwin W.R., Urban R.R. (2021). Pembrolizumab with low-dose carboplatin for recurrent platinum-resistant ovarian, fallopian tube, and primary peritoneal cancer: survival and immune correlates. J Immunother Cancer.

[176] Matulonis U.A., Shapira-Frommer R., Santin A.D. (2019). Antitumor activity and safety of pembrolizumab in patients with advanced recurrent ovarian cancer: results from the phase II KEYNOTE-100 study. Ann Oncol.

[177] Konstantinopoulos P.A., Waggoner S., Vidal G.A. (2019). Single-arm phases 1 and 2 trial of niraparib in combination with pembrolizumab in patients with recurrent platinum-resistant ovarian carcinoma. JAMA Oncol.

[178] Balar A.V., Weber J.S. (2017). PD-1 and PD-L1 antibodies in cancer: current status and future directions. Cancer Immunol Immunother.

[179] Zhang L., Chen Y., Li F., Bao L., Liu W. (2019). Atezolizumab and bevacizumab attenuate cisplatin resistant ovarian cancer cells progression synergistically via suppressing epithelial-mesenchymal transition. Front Immunol.

[180] Lee E.K., Xiong N., Cheng S.C. (2020). Combined pembrolizumab and pegylated liposomal doxorubicin in platinum resistant ovarian cancer: a phase 2 clinical trial. Gynecol Oncol.

[181] Zsiros E., Lynam S., Attwood K.M. (2021). Efficacy and safety of pembrolizumab in combination with bevacizumab and oral metronomic cyclophosphamide in the treatment of recurrent ovarian cancer: a phase 2 nonrandomized clinical trial. JAMA Oncol.

[182] Walsh C.S., Kamrava M., Rogatko A. (2021). Phase II trial of cisplatin, gemcitabine and pembrolizumab for platinum-resistant ovarian cancer. PLoS One.

[183] Pujade-Lauraine E., Fujiwara K., Ledermann J.A. (2021). Avelumab alone or in combination with chemotherapy versus chemotherapy alone in platinum-resistant or platinum-refractory ovarian cancer (JAVELIN Ovarian 200): an open-label, three-arm, randomised, phase 3 study. Lancet Oncol.

[184] Lee J.Y., Kim B.G., Kim J.W. (2022). Biomarker-guided targeted therapy in platinum-resistant ovarian cancer (AMBITION; KGOG 3045): a multicentre, open-label, five-arm, uncontrolled, umbrella trial. J Gynecol Oncol.

[185] Wang Z., Meng F., Zhong Z. (2021). Emerging targeted drug delivery strategies toward ovarian cancer. Adv Drug Deliv Rev.

[186] Miller E.M., Samec T.M., Alexander-Bryant A.A. (2021). Nanoparticle delivery systems to combat drug resistance in ovarian cancer. Nanomedicine.

[187] Mensah L.B., Morton S.W., Li J. (2019). Layer-by-layer nanoparticles for novel delivery of cisplatin and PARP inhibitors for platinum-based drug resistance therapy in ovarian cancer. Bioeng Transl Med.

[188] Zhang X., Liu Y., Kim Y.J., Mac J., Zhuang R., Wang P. (2017). Co-delivery of carboplatin and paclitaxel *via* cross-linked multilamellar liposomes for ovarian cancer treatment. RSC Adv.

[189] Yellepeddi V.K., Kumar A., Maher D.M., Chauhan S.C., Vangara K.K., Palakurthi S. (2011). Biotinylated PAMAM dendrimers for intracellular delivery of cisplatin to ovarian cancer: role of SMVT. Anticancer Res.

[190] Zhang M., Hagan C.T., Min Y. (2018). Nanoparticle co-delivery of wortmannin and cisplatin synergistically enhances chemoradiotherapy and reverses platinum resistance in ovarian cancer models. Biomaterials.

[191] Zheng W., Li M., Lin Y., Zhan X. (2018). Encapsulation of verapamil and doxorubicin by MPEG-PLA to reverse drug resistance in ovarian cancer. Biomed Pharmacother.

[192] Bayda S., Hadla M., Palazzolo S. (2018). Inorganic nanoparticles for cancer therapy: a transition from lab to clinic. Curr Med Chem.

[193] Song H., Quan F., Yu Z. (2019). Carboplatin prodrug conjugated Fe_3_O_4_ nanoparticles for magnetically targeted drug delivery in ovarian cancer cells. J Mater Chem B.

[194] Shahin S.A., Wang R., Simargi S.I. (2018). Hyaluronic acid conjugated nanoparticle delivery of siRNA against TWIST reduces tumor burden and enhances sensitivity to cisplatin in ovarian cancer. Nanomed Nanotechnol Biol Med.

[195] Franke C.E., Czapar A.E., Patel R.B., Steinmetz N.F. (2018). Tobacco mosaic virus-delivered cisplatin restores efficacy in platinum-resistant ovarian cancer cells. Mol Pharm.

[196] Zhang X., Liu L., Tang M., Li H., Guo X., Yang X. (2020). The effects of umbilical cord-derived macrophage exosomes loaded with cisplatin on the growth and drug resistance of ovarian cancer cells. Drug Dev Ind Pharm.

[197] Kawahara B., Ramadoss S., Chaudhuri G., Janzen C., Sen S., Mascharak P.K. (2019). Carbon monoxide sensitizes cisplatin-resistant ovarian cancer cell lines toward cisplatin via attenuation of levels of glutathione and nuclear metallothionein. J Inorg Biochem.

[198] Serini S., Cassano R., Bruni M., Servidio C., Calviello G., Trombino S. (2021). Characterization of a hyaluronic acid and folic acid-based hydrogel for cisplatin delivery: antineoplastic effect in human ovarian cancer cells *in vitro*. Int J Pharm.

[199] Ambrosio A.J., Suzin D., Palmer E.L., Penson R.T. (2014). Vintafolide (EC145) for the treatment of folate-receptor-α positive platinum-resistant ovarian cancer. Expet Rev Clin Pharmacol.

